# Cost optimization for flexible pavement on fine sand improved using palm fibers

**DOI:** 10.1038/s41598-025-02115-7

**Published:** 2025-05-20

**Authors:** Ahmed H. Elbosraty, M. Bahr, Ahmed M. Ebid

**Affiliations:** 1 Department of Civil Engineering, Al-Azhar University in Egypt, Cairo, Egypt; 2 Department of Structural Engineering and Construction Management, Future University in Egypt, Cairo, Egypt

**Keywords:** CBR, Fine sand, Palm fiber, Flexible pavement, Urban roads, Rural roads, Cost optimization, Civil engineering, Sustainability

## Abstract

Improving the CBR value of the fine sand sub-soil layer is critical for enhancing flexible pavement roads’ stability and cost efficiency. This study investigates the impact of mixing fine sand with palm fibers on its CBR value in order to minimize the total cost. Experimental tests were conducted considering different palm fiber contents (0.5%, 1.0%, and 1.5% by weight), and the results indicate that the maximum CBR value of 25% was archived using palm fiber content of 1.0%. Beyond this content, a decline in CBR was noted due to fiber clustering. The AASHTO Pavement Design Guide was used to investigate the impact of enhancing the sub-soil CBR value on the thickness and the cost of the base and sub-base layers for both urban and rural roads. The results showed that increasing the CBR value reduces both the thickness and the costs of these layers. In addition, the option of using a sub-base layer is more cost effective for rural roads on weak soil (CBR < 20%) and for high-traffic urban roads. Finally, a cost analysis study was conducted to optimize the total cost of soil improvement and (base & sub-base) layers. The outcomes indicated that the cost-effective CBR values are ranged between (13% & 17%) for rural roads and (17% and 19%) for urban roads. These values are equivalent to palm fiber content of (0.17% & 0.33%) for rural roads and (0.33% & 0.43%) for urban roads. Although this study showed that using organic reinforcement like palm fibers is a sustainable, economical, and eco-friendly solution, its durability and environmental degradation should be investigated.

## Introduction

Pavement design ensures durability, safety, and cost efficiency in transportation infrastructure. Flexible pavements, characterized by their multi-layered structure, require precise design considerations to withstand traffic loads and environmental factors. This study focuses on the effect of subgrade soil properties, particularly California Bearing Ratio (CBR), on pavement design^1–5^. Soil improvement is an important area in geotechnical engineering that aims to improve the physical, chemical and mechanical properties of soils to meet specific technical requirements. Previous studies have examined a few processes and materials soil quality and its impact on CBR values^6–8^. The CBR test is widely used to assess the load-bearing capacity of subgrade soils. Numerous studies have explored various methods and materials for soil improvement. For instance, Singh et al. demonstrated significant improvements in CBR values with the addition of natural fibers like jute fiber^9^, while Jamshidi Chenari et al. highlighted the benefits of using expanded polystyrene (EPS) combined with binders such as cement and fly ash to enhance the mechanical properties of sandy soils^10^. Similarly, Chegenizadeh et al. and Zadhoush, et al. found that both natural and synthetic fibers significantly increased the CBR values of silty sand soils^11,12^.

Recent studies have emphasised the combination of superior experimental and analytical techniques to improve pavement overall performance and sustainability. Research on geopolymer concrete mixes for high-traffic roads highlights the position of eco-friendly substances in enhancing mechanical residences and durability. Accelerated checking out and climate-aware machine learning models similarly aid predictive upkeep and performance assessment. Moreover, cost estimation procedures are evolving with the utility of life-cycle cost analysis and AI-based modeling. Studies utilising AASHTOWare and image processing strategies show the developing reliance on digital tools for pavement deterioration forecasting and decision-making. These present day strategies provide extra reliable statistics for long-term infrastructure planning^13–18^.

The effect of palm fiber reinforcement on the mechanical behavior of rammed earth was studied in some studies, where different fiber contents (0.5%, 1%, and 1.5%) were incorporated, and tests were conducted to assess their impact on moisture content, density, compressive strength, and tensile properties. The results indicate that fiber reinforcement enhances both compressive and tensile strength, with an optimal fiber content identified^19–21^.

The design of flexible pavements is intricately linked to subgrade quality, where enhancements using recycled materials like plastic waste have been shown to optimize costs. Emphasized optimizing subgrade treatment depth to improve pavement performance while reducing asphalt thickness^22^. Sustainable solutions have also gained attention, such as using rice husk ash and other recycled materials, which improve subgrade performance and contribute to environmental sustainability^23^.

Artificial intelligence (AI) and machine learning have significantly progressed pavement engineering, improved soil stabilization, and predicted subgrade and subbase performance. Research has shown the accuracy of AI models, including artificial neural networks (ANN), genetic programming (GP), and evolutionary polynomial regression (EPR), in predicting the CBR and unconfined compressive strength (UCS) of treated soils. These methodologies ensure elevated precision and facilitate effective decision-making in pavement construction and maintenance^24–29^.

Palm fiber, an abundant agricultural byproduct in many tropical and subtropical regions, particularly in the Middle East and North Africa, offers a sustainable and low-cost reinforcement alternative for geotechnical applications. It possesses high tensile strength and biodegradability, making it suitable for improving soil mechanical behavior. While past research has addressed the mechanical enhancement of soils with palm fiber, limited studies have explored its specific impact on fine sand or linked this to cost optimization in pavement design. This study fills this research gap by evaluating the effect of palm fiber content on soaked CBR and correlating these improvements with reductions in pavement layer thickness and overall construction cost.

Palm fibers are a byproduct of agricultural processing and are widely available in palm-growing regions such as Egypt, Saudi Arabia, and other parts of the Middle East and North Africa. Their availability in large quantities, combined with their low cost and biodegradability, makes them a practical candidate for large-scale applications in road construction projects, particularly in rural and developing areas where such materials are often discarded as waste.

Furthermore, various soil stabilizing techniques, such as Ashcrete, nanocomposite binders, and diverse additives, have been investigated to enhance soil characteristics. An integrated decision support system (DSS) utilizing value engineering (VE) and the Analytical Hierarchy Process (AHP) has demonstrated efficacy in optimizing soil treatment options for highway construction. Combining AI with sustainable materials and decision support systems facilitates the development of more resilient and economical pavement solutions^30,31^.

Although several studies have reported the mechanical benefits of using natural fibers in soil reinforcement, most have not evaluated the cost-effectiveness of these improvements within the framework of established pavement design methods. Furthermore, very limited research has focused on palm fiber applications in fine sand, particularly regarding the correlation between fiber dosage, CBR value, and its influence on pavement layer design and construction cost. This study aims to fill this gap by providing a quantitative analysis that integrates material testing with pavement design optimization and cost modeling.

This research aims to evaluate pavement performance and associated costs under varying CBR values, considering the presence or absence of a sub-base layer. Additionally, the research explores innovative soil improvement techniques using cost-effective materials to enhance the load-bearing capacity of the subgrade.

## Methodology

**Fig. 1:**
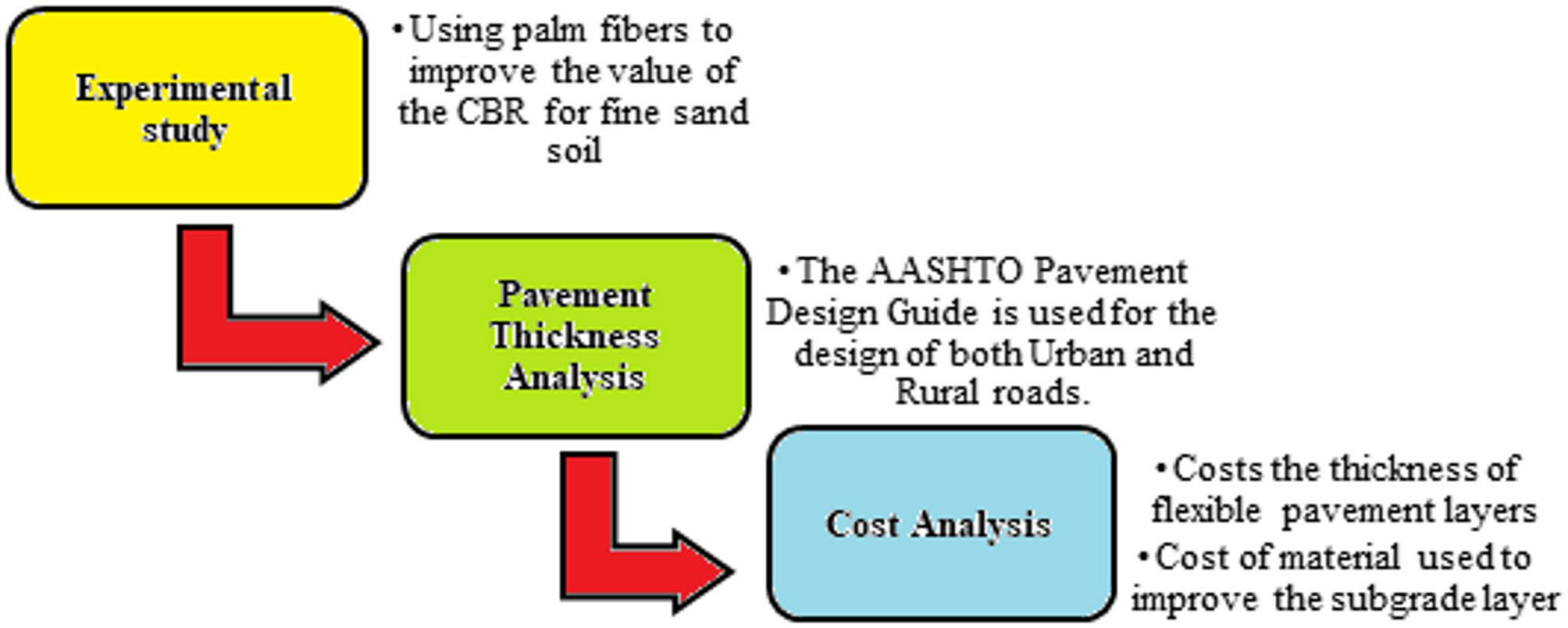
Research methodology.

To achieve the study objectives, three-phases methodology was considered. The first phase was dedicated to experimentally investigating the enhancement in fine sand’s CBR value due to adding different doses of palm fibers and to present this enhancement as a mathematical formula. While the second phase was concerned in studying the impact of changing the CBR value of the sub-soil layer on the structural design of flexible pavement of both urban and rural roads (layers thicknesses). Finally, the third phase was meant to estimate the total cost of road (soil improvement + pavement) to determine the optimum palm fiber content and the corresponding CBR value. Figure 1 presents the considered methodology. The considered three phases are described in details in the following paragraphs.

- Phase 1: Experimental Testing: Four soil samples were prepared by mixing fine sand with varying contents of palm fiber: 0.0%, 0.5%, 1.0%, and 1.5% by weight. The fibers used were oven-dried and manually shredded before mixing. Each sample was compacted and tested following the AASHTO T193 (soaked CBR test method). For accuracy, each test was conducted in triplicate, and the average value with standard deviation was reported. The compaction was done using a modified Proctor hammer to achieve uniform density. All CBR tests were conducted under soaked conditions as per AASHTO T193 standards, which are recommended for pavement design evaluations. The palm fibers used in this study were oven-dried at 60 °C for 24 h and manually shredded before mixing. No chemical treatment was applied, as the aim was to evaluate the effectiveness of untreated natural fibers.
- Phase 2: Pavement Design: The AASHTO 1993 Pavement Design Guide was used to assess the impact of subgrade CBR improvements on the required thickness of the base and sub-base layers for both rural and urban roads. Design inputs included ESALs, reliability, drainage coefficient, material moduli, and layer coefficients based on standard values. The 1993 AASHTO Guide was used in this study due to its widespread adoption in current practice, especially in developing countries. While it provides reasonable accuracy for structural design, it is acknowledged that mechanistic-empirical approaches offer more comprehensive assessments and will be considered in future phases of this research.
- Phase 3: Cost Analysis: The cost of soil improvement (based on the weight and unit cost of palm fiber) and pavement layers (base and sub-base) was calculated. The total cost per square meter of road was then estimated under different CBR values, and the optimum CBR range was identified by evaluating cost trends.

## Experimental study

**Fig. 2:**
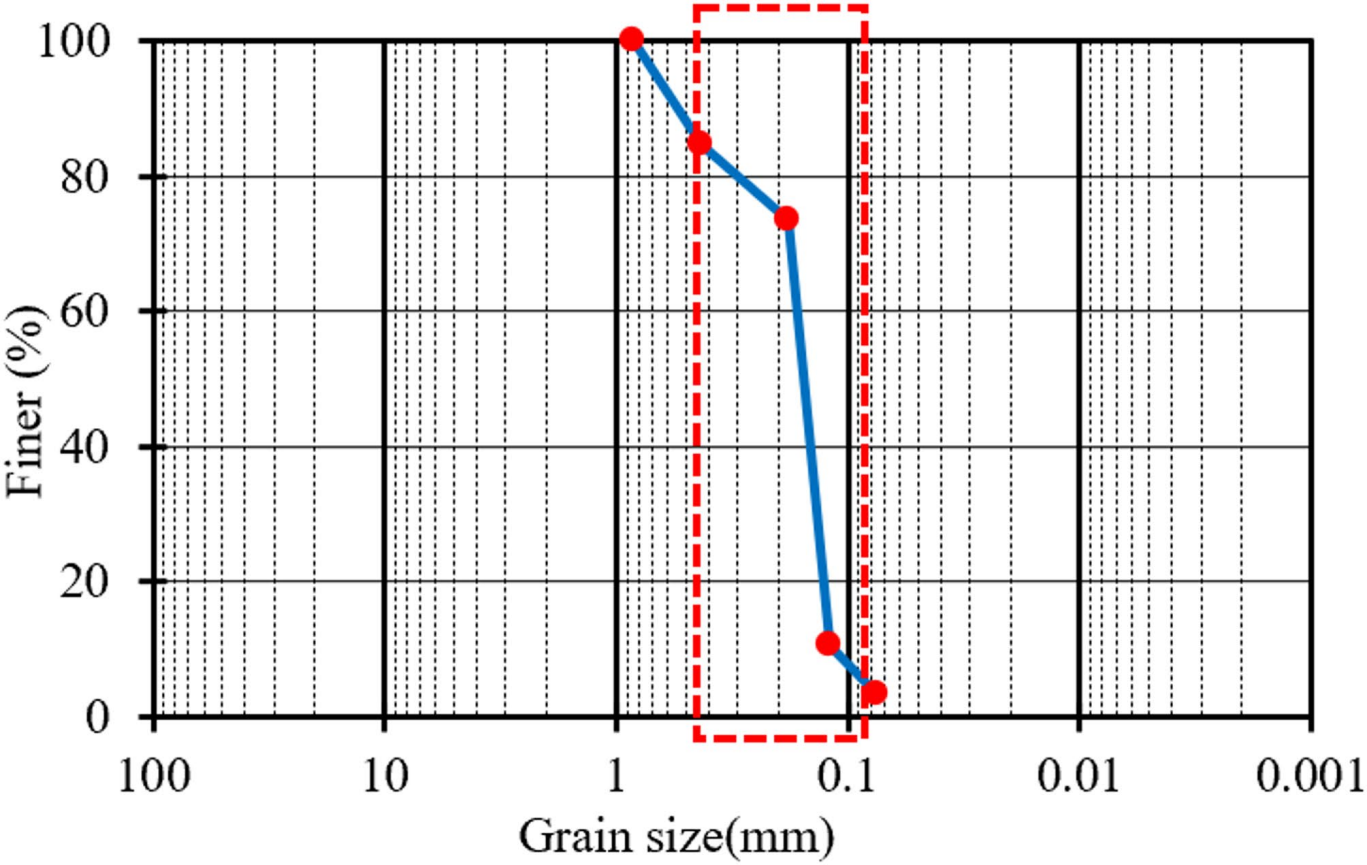
Grain size distribution of the tested soil showing that the majority of particles fall within the fine sand range (0.075–0.425 mm) as per ASTM D2487 classification.

**Fig. 3:**
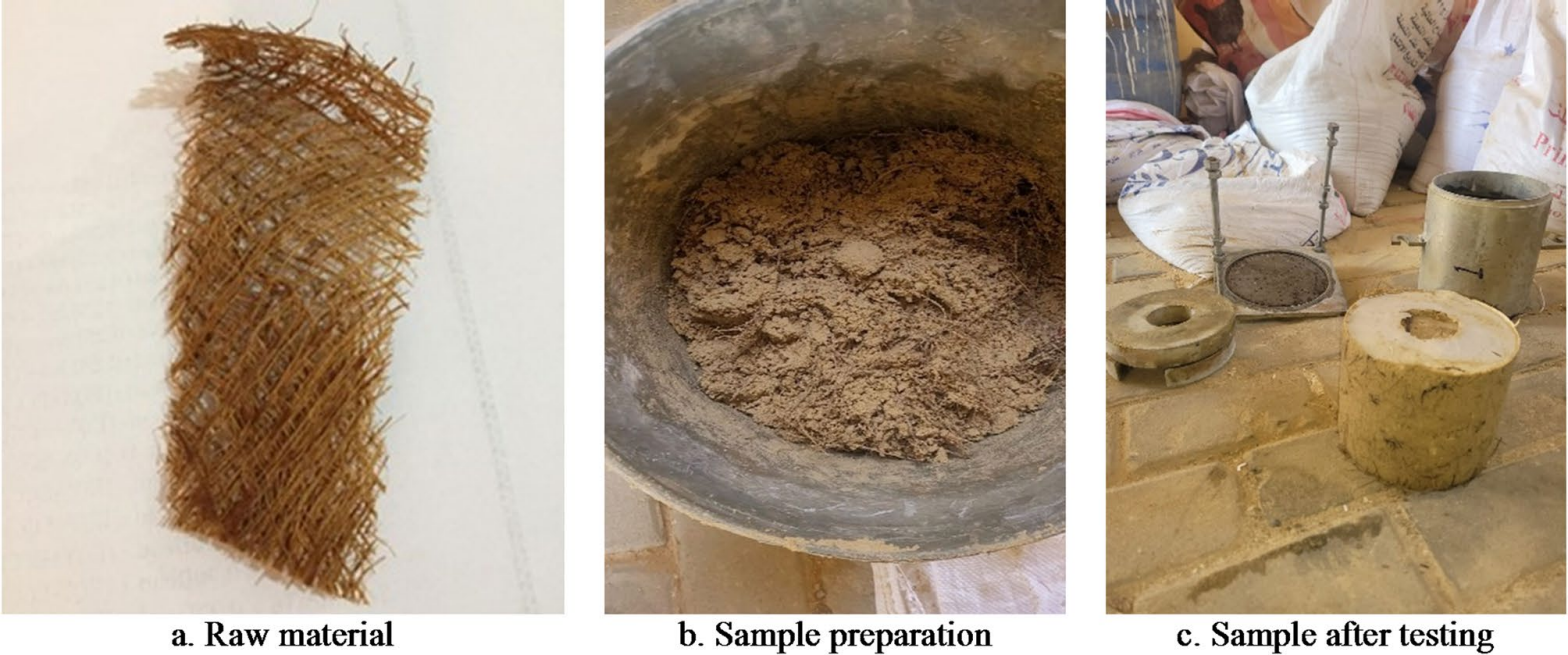
Experimental study process.

This phase aims to experimentally investigate the impact of adding different doses of palm fibers to the fine sand on its CBR value. In order to achieve this aim, four fine sand samples were mixed with different amounts of palm fibers (0.0%, 0.5%, 1.0% & 1.5% by weight) and tested according to AASHTO T180/ASTM D1557 to determine their soaked CBR values, as shown in Table 1. Figure 2, presents the grain size analysis of the considered fine sand sample, while Fig. 3 shows the test process. The tested soil was classified as fine sand according to ASTM D2487, with the majority of particles falling between 0.075 mm and 0.425 mm, as confirmed in the grain size distribution. The CBR values reported in this study are soaked values, reflecting field conditions for flexible pavement design. Each CBR test was conducted three times (triplicate testing) for each fiber content level. The average CBR value was reported along with its standard deviation to ensure the reliability and consistency of the test results.

**Table 1:** Results of adding the palm fiber/weight.

% of palm fiber/weight	CBR (%)	Standard deviation (%)
0%	9%	± 0.5
0.5%	20%	± 0.6
1%	25%	± 0.4
1.5%	22%	± 0.5

**Fig. 4:**
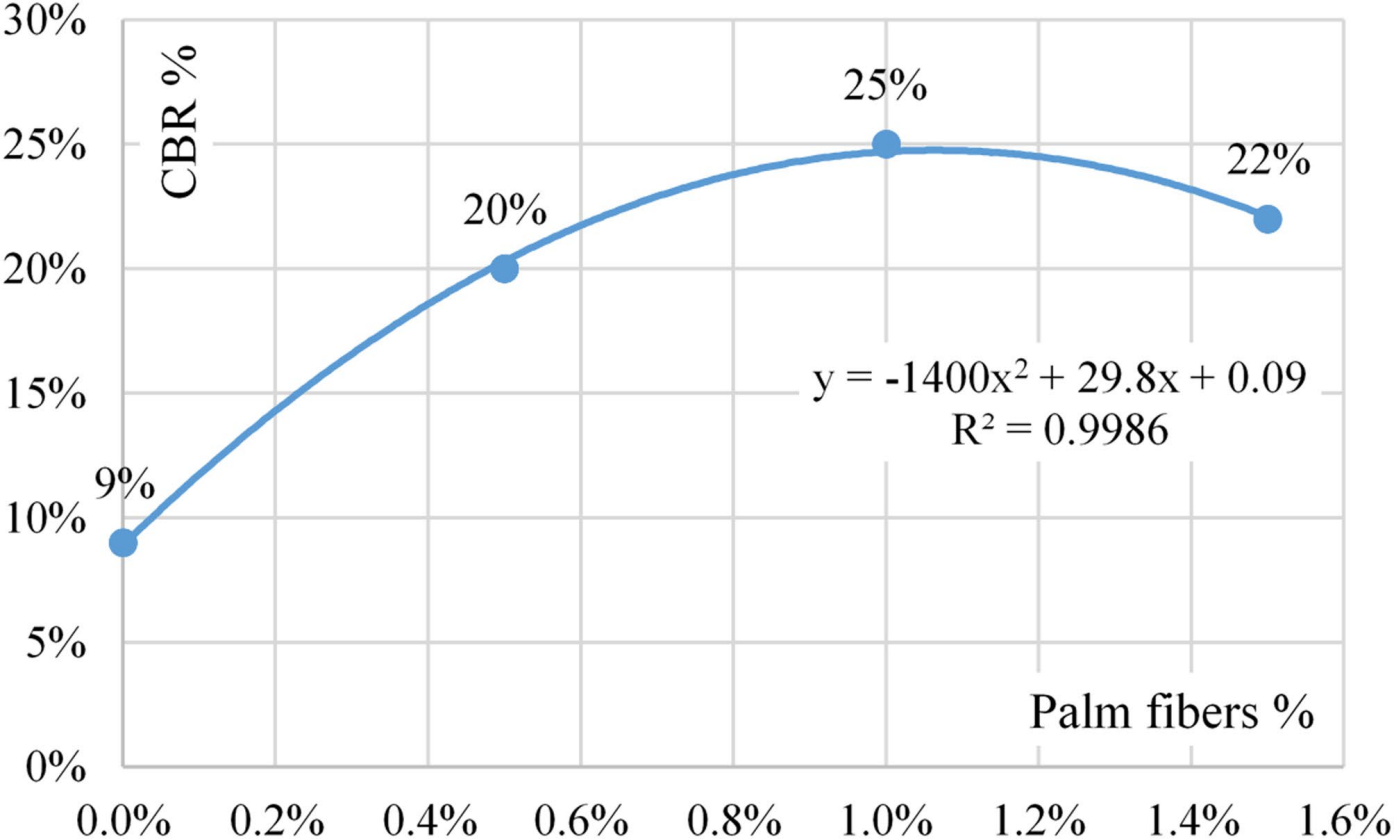
Relation between palm fiber content and CBR values.

Figure 4 shows the relation between palm fiber content and CBR values. The best-fitting formula is presented in Eq. 1. Finally, Table 2 summarizes the corresponding palm fiber content and its cost for different CBR values (form 9% and up to 25%) based on Eq. 1. The amount and the cost of palm fibers per cubic meter of soil was calculated considering the compacted density of fine sand equals to 1600 kg/m3. The palm fibers’ price equals 350$/ton as per (www.globalsources.com) in 2025.

All samples were prepared by oven-drying both the fine sand and palm fibers to ensure consistency in moisture content. Palm fibers were shredded manually and uniformly mixed with the sand. The modified Proctor method was used to compact each sample at its optimum moisture content. To minimize variability, all samples were tested under the same temperature and humidity conditions in a controlled lab environment. For each fiber content, three replicates were tested, and average CBR values with standard deviations were reported.

The optimal fiber content was not selected based solely on the highest experimental CBR. Instead, a second-order regression model (Eq. 1) was used to identify the peak performance range, which was then verified by testing additional intermediate fiber contents (0.75% and 1.25%) as shown in Table 3.

1 $${\text{CBR }} = {\text{ }} - {\text{14}}00\left( {{\text{Palm fiber}}} \right)^{{\text{2}}} + {\text{ 29}}.{\text{8}}\left( {{\text{Palm fiber}}} \right){\text{ }} + {\text{ }}0.0{\text{9}}$$

To validate the accuracy of the empirical model (Eq. 1), two additional CBR tests were performed at intermediate fiber contents of 0.75% and 1.25%. The experimental values closely matched the predicted CBR values from the equation, with errors less than 5%. This supports the reliability of the regression model within the tested range, as shown in Table 3.

**Table 2:** Palm fiber content and corresponding cost and CBR value.

CBR %	9%	11%	13%	15%	17%	19%	21%	23%	25%
Palm fiber %	0.00	0.10%	0.17%	0.25%	0.33%	0.43%	0.55%	0.70%	1.00%
Palm fiber content (kg/m3)	0.0	1.6	2.7	4.0	5.3	6.9	8.8	11.2	16.0
Palm fiber cost ($/m3)	0.00	0.56	0.95	1.40	1.85	2.41	3.08	3.92	5.60

**Table 3:** Verification of empirical model (equation 1) with intermediate palm fiber contents.

Palm fiber (%)	Experimental CBR (%)	Predicted CBR (%)	Error (%)
0.75	23.4	22.9	2.1
1.25	23	22.1	3.9

## Pavement design

The main goal of this phase is to estimate the reduction in the pavement layers by increasing the CBR value of sub-soil. Accordingly, The AASHTO Pavement Design Guide was used to calculate the thicknesses of pavement layers considering different road configurations and different values for sub-soil CBR as follows:

- The equivalent single axle loads (ESALs) considering 2% annual growth rate for 15 years is:
  - 10 million for urban roads.
  - 5 million for rural roads.
- Two scenarios were studied,
  - Using only base layer (6” Asphalt + variable thickness base layer).
  - Using base and sub-base layers (6” Asphalt + 6” base layer + variable thickness sub-base layer).
- Finally, all scenarios were designed considering sub-soil CBR values of (9, 11, 13, 15, 17, 19, 21, 25%).

**Fig. 5:**
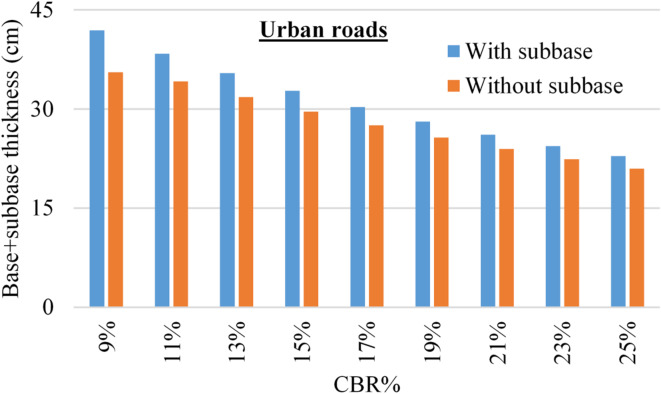
The thickness of flexible pavement layers, with and without sub-base layer for urban roads, for different CBR values.

**Fig. 6:**
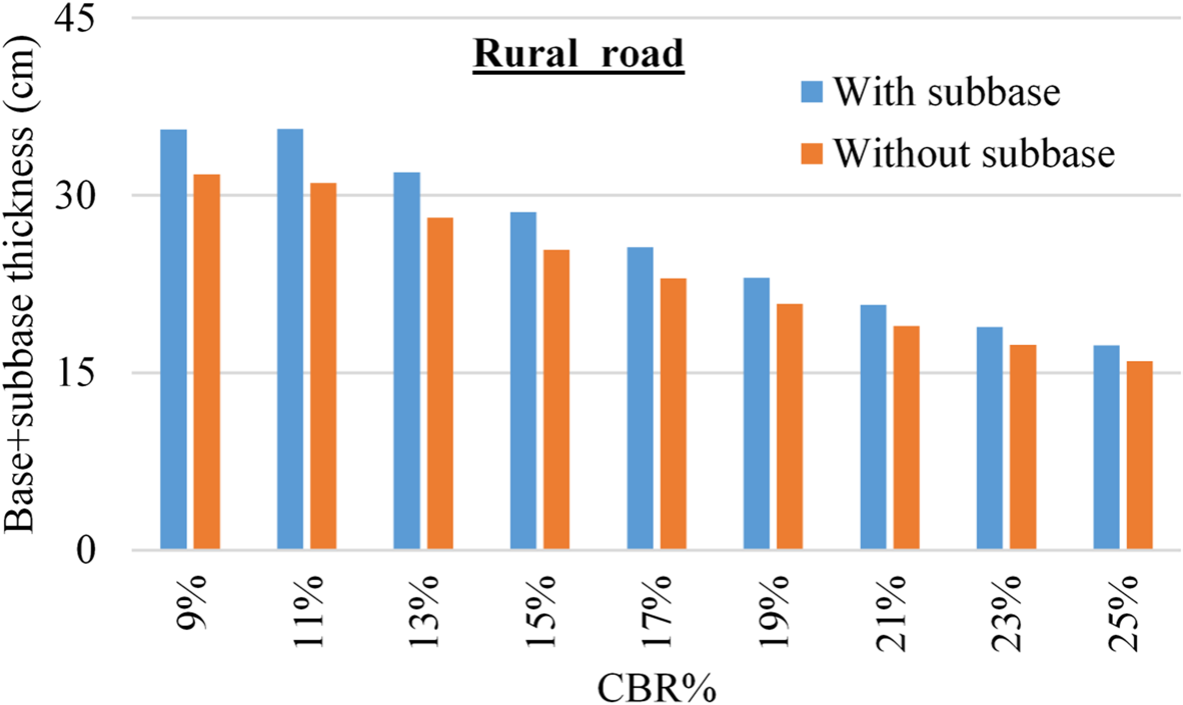
Variation in total pavement thickness for rural roads using different subgrade CBR values, with and without sub-base layers.

The considered design parameters for all cases are listed in Table 4 and all calculations were conducted using the online design tool (https://pavementinteractive.org/apps/calculators/1993-aashto-flexible-pavement-structural-design/ ). Figures 5 and 6 summarized the designed (base + sub base) thickness for both urban and rural roads.

**Table 4:** The considered pavement design parameters.

Parameter	Unit	Value
Standard deviation (S_0_)	–	0.45
The reliability (R) value	–	95%
The drainage coefficient	m	1.15
Initial serviceability index (Pi)	–	4
Terminal serviceability index (Pt)	–	2.5
Resilient modulus (MR) for asphalt	Psi	430,000
MR for the base (CBR = 80%)	Psi	52,000
MR for the sub-base (CBR = 50%)	Psi	38,000
Asphalt layer coeff. (a1)	–	0.420
Base layer coeff. (a2)	–	0.120
Sub-base layer coeff. (a3)	–	0.095

**Fig. 7:**
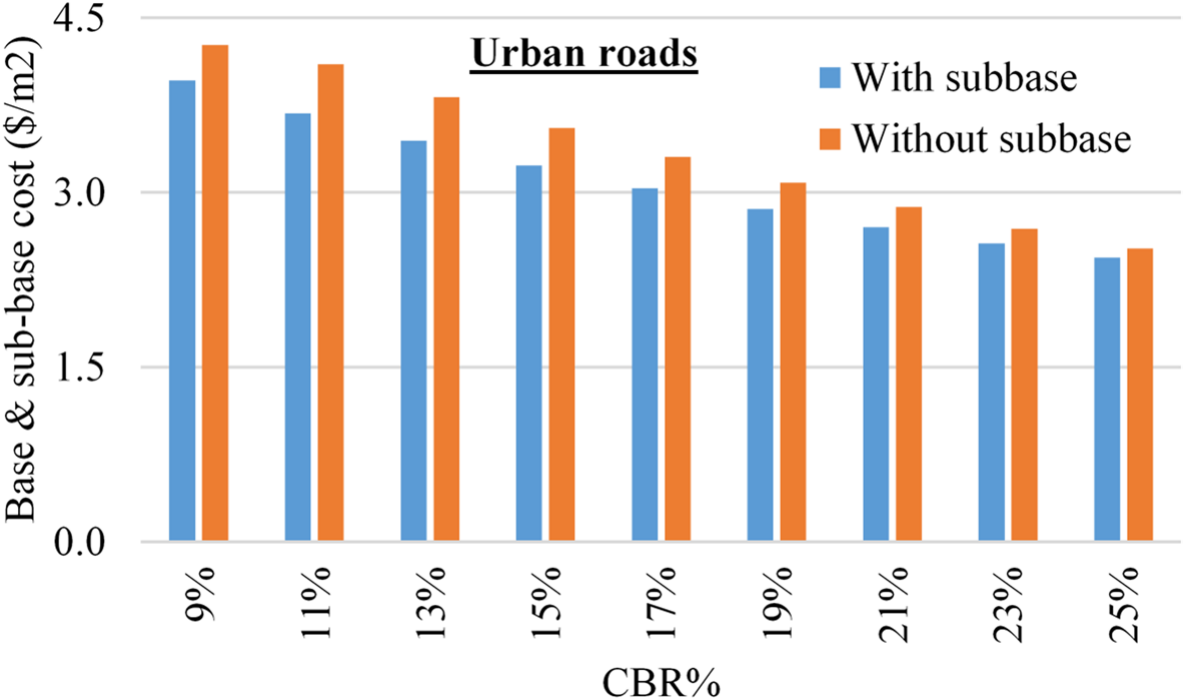
The cost of base & sub-base layers, for urban roads, using different CBR values.

**Fig. 8:**
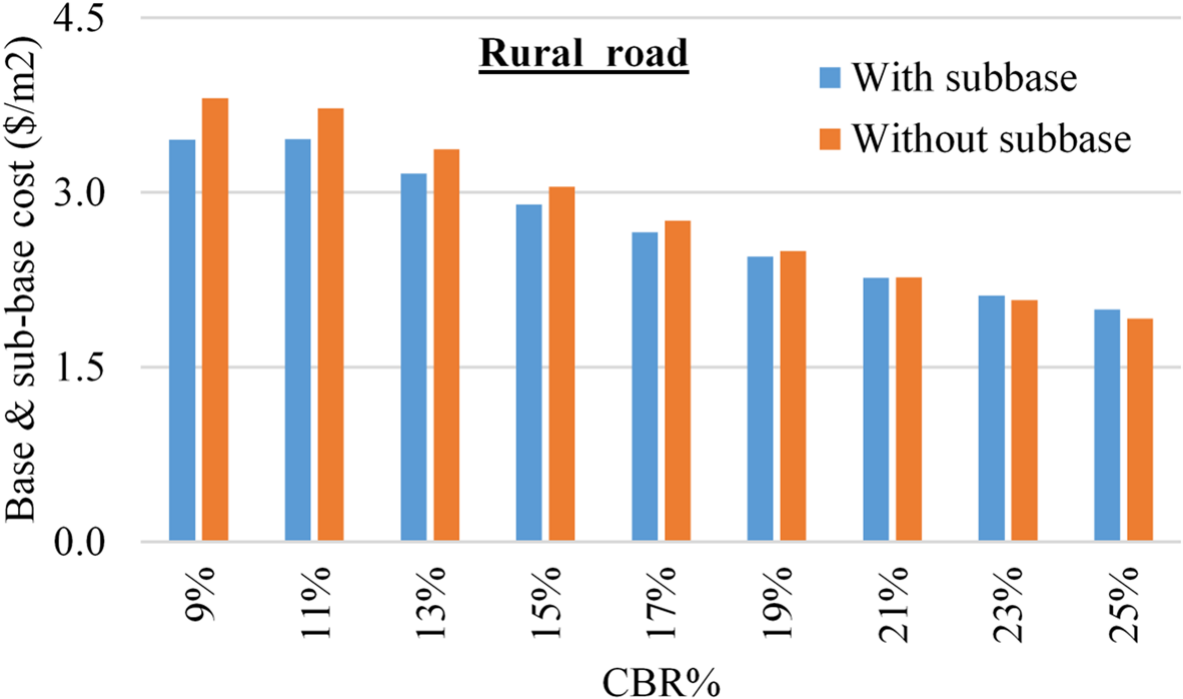
The cost of base & sub-base layers for rural roads, using different CBR values.

The total cost of the base and sub-base layers was calculated for each sub-soil CBR value considering (12$/m3) for the base layer and (8$/m3) for the sub-base layer, after (https://aggregatemarkets.com). Figures 7 and 8 summarize the base & sub-base costs considering different CBR values for urban and rural roads.

## Cost analysis

**Fig. 9:**
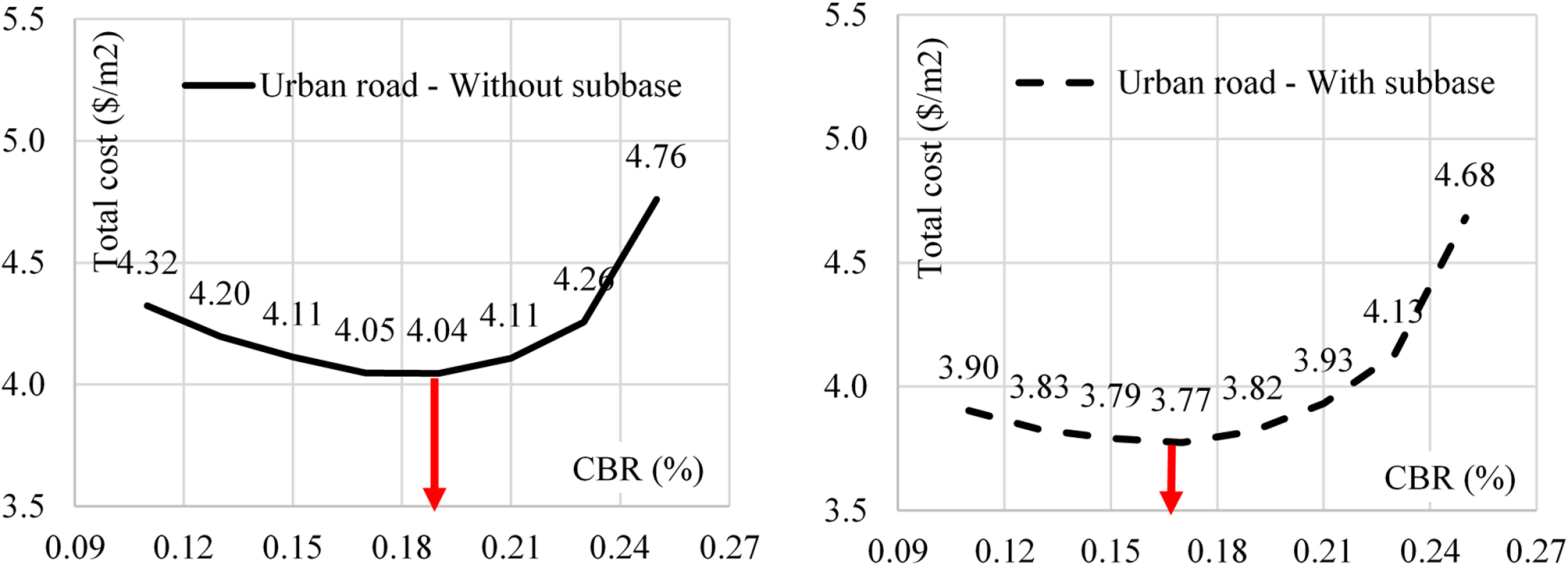
The total cost Vs CBR value for urban road with and without sub-base.

**Fig. 10:**
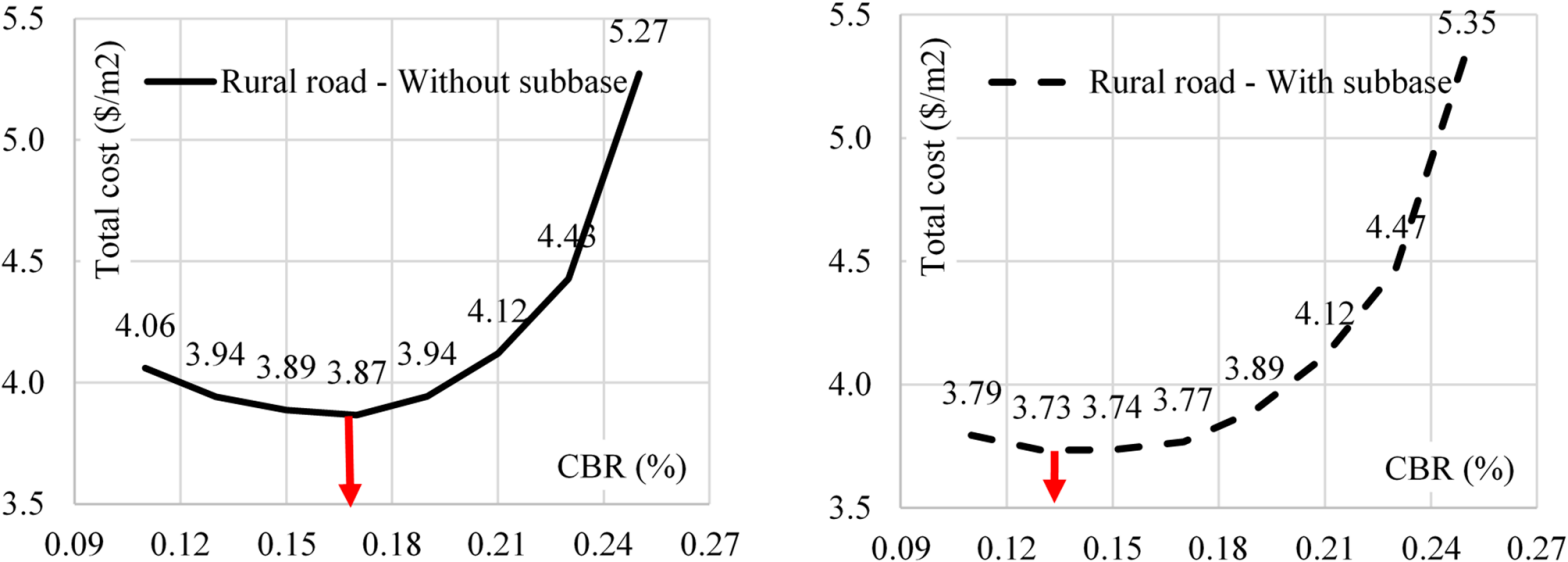
The total cost Vs CBR value for rural road with and without sub-base.

During this phase, the total cost of base & sub-base layers was combined with the cost of the sub-soil improvement using palm fibers. As AASHTO Pavement Design Guide recommended, the improved sub-soil depth was considered 0.6 m for urban roads and 0.4 m for rural roads. Accordingly, the total cost of base, sub-base and sub-soil improvement was calculated using different sub-soil CBR values for urban and rural roads. Figures 9 and 10 illustrate these results.

Cost breakdown summary:

- Palm fiber cost was calculated using a market rate of $350/ton and a soil density of 1600 kg/m^3^.
- The required fiber mass per cubic meter was derived from the percentage content and then multiplied by the fiber unit cost.
- Pavement layer costs were calculated based on required thicknesses obtained from the AASHTO design for each CBR level:
  - Base layer cost = thickness × area × $12/m^3^.
  - Sub-base layer cost = thickness × area × $8/m^3^.
- The total cost per square meter was the sum of subgrade treatment and layer material costs for each CBR value.

Although this study focused on the initial construction cost, it is important to consider lifecycle costs, including periodic maintenance and repair. Improved subgrade performance may reduce long-term maintenance needs, potentially leading to additional savings. Future research should incorporate a life-cycle cost analysis (LCCA) to evaluate the full economic benefits of palm fiber-reinforced pavement systems.

## Discussion

Revising the results of the experimental CBR tests indicated that the CBR value increased from 9 to 25% with increasing the palm fiber content from 0.0 to 1.0% (by weight); beyond this limit, the CBR decreased to 22% at the palm fiber content of 1.5%. The CBR value improved due to the tensile strength of the added palm fibers, which increased the sample’s shear strength, while the reason of the CBR reduction beyond the optimum palm fiber content (1.0%) is the fiber clustering which caused a reduction in compaction efficiency and hence shear strength. Accordingly, using palm fiber content beyond the optimum limit is not recommended to improve the fine sand layers. These results matched the previously published works^6,12^.

Regarding the flexible pavement design, the illustrated results in Figs. 5 and 6 indicated that for both urban and rural roads, the total thickness of base and sub-base layers decreases by increasing the sub-soil CBR value due to the enhancement of the shear strength. In addition, it could be noted that in all cases, the total thickness of (base & sub-base) option is always more than the total thickness of (base only) option, and the ratio between these two total thicknesses decreased from (112–108%) for rural roads, and from (118–109%) for urban roads with increasing the CBR value from (9–25%).

Figures 7 and 8 showed that in all cases, the cost of base and sub-base layers decreased with increasing the CBR value of the sub-soil (due to decreasing their thicknesses). For urban roads, the (sub-base) option is always cheaper than (base only) option, while the difference between the two costs decreased from (7–3%) with increasing the CBR value from (9–25%). Similarly, for rural roads, is cheaper than the (base only) option up to CBR value of 21%, beyond this limit, the (base only) option become cheaper than (sub-base) option. The ratio of (sub-base)/(base only) costs increases from (91–104%) with increasing the CBR from (9–25%).

The relations between the total cost of soil improvement and (base & sub-base) layers verses the sub-soil CBR values are presented in Figs. 9 and 10. Both figures showed that the total cost decreased with increasing the CBR value up to optimum value (depends on the road type and the pavement option) and increased beyond this limit.

The observed optimum CBR values are (13% & 17%) for rural roads using (sub-base) & (base only) options respectively. The optimum CBR valued for urban roads are (17% & 19%) for (sub-base) & (base only) options respectively. From Table 2, these optimum CBR values are equivalent to optimum palm fiber contents of (0.17% & 0.33%) and (0.33% & 0.43%) for rural and urban roads in order.

The increase in CBR up to 1.0% palm fiber content is attributed to the fibers’ ability to bridge soil particles, enhance interlocking, and resist shear deformation. However, when the fiber content exceeds this optimum threshold, the fibers begin to cluster and entangle, leading to void formation and reduced compaction effectiveness. This clustering effect negatively impacts the load-bearing capacity of the soil, thereby reducing the CBR value. Similar behavior has been reported in studies by Hejazi et al.^12^ and Arabani et al.^13^.

## Conclusions, limitations, and future works

This study investigated the impact of mixing fine sand with palm fibers to improve its CBR value and optimize the cost of flexible pavement construction. The experimental tests results showed a significant improvement in the (CBR) of fine sand. The maximum achieved CBR value was 25% at fiber content of 1.0%. This enhancement reduced pavement cost by reducing the required thickness of base and sub-base layers. In addition, the organic nature of palm fiber makes this mixture a sustainable, economical and eco-friendly pavement solution.

Additionally, the research indicated that using a sub-base layer reduces the pavement cost, especially for high-traffic urban roads and rural roads on weak soil (CBR < 20%). The cost analysis results showed that the optimum CBR values to minimize the cost are 13% and 17% for rural roads (with sub-base and base-only options, respectively) and 17% and 19% for urban roads under similar configurations.

Accordingly, it is not a cost-effective decision to use 1% palm fiber content to achieve the maximum CBR value of 25%, but the wise decision is to use palm fiber content between (0.17% and 0.43%) depending on the traffic load and the pavement layers to minimize the total cost.

This study’s outcomes did not consider palm fiber’s long-term performance under environmental conditions. Accordingly, future research is recommended to study the durability and environmental degradation of palm fibers and their impact on the stability and settlements of roads.

The palm fibers used were untreated and biodegradable, which may affect long-term performance. Future studies are recommended to evaluate chemically treated fibers and assess their durability under real environmental conditions.

The study was limited to the evaluation of soaked CBR values to assess the applicability of palm fiber-reinforced fine sand in flexible pavement systems. Although this aligns with the AASHTO design framework, other mechanical properties such as unconfined compressive strength (UCS), tensile strength, and modulus of resilience were not investigated. In addition, the long-term performance and durability of palm fibers under varying moisture and environmental exposure remain unexamined. Future research should incorporate these parameters to provide a more comprehensive understanding of the material’s behavior.

This study focused exclusively on the CBR test, which is a key parameter in flexible pavement design. However, it is recommended that future research investigate additional engineering properties such as UCS, resilient modulus (MR), and compaction characteristics to provide a more complete evaluation of the mechanical performance of fiber-reinforced soils.

Future work should include the use of the Mechanistic-Empirical Pavement Design Guide (MEPDG) to validate and potentially refine the design implications derived from the current analysis.

## Data Availability

All the generated data are summarized in Table 2.
